# Importance of Patient-Derived Xenograft Models in Battling Cancer Therapy Resistance

**DOI:** 10.3390/cancers18142187

**Published:** 2026-07-08

**Authors:** Ákos Juhász, Sára Eszter Surguta, Laura Svajda, Ivan Ranđelović, Andrea Ladányi, József Tóvári, Mihály Cserepes

**Affiliations:** 1 Pharmacy, National Institute of Oncology, 1122 Budapest, Hungary; 2 Doctoral College, Semmelweis University, 1085 Budapest, Hungary; 3 Department of Experimental Pharmacology and the National Tumor Biology Laboratory, National Institute of Oncology, 1122 Budapest, Hungary; 4 KINETO Lab Ltd., 1037 Budapest, Hungary; 5 Department of Surgical and Molecular Pathology and the National Tumor Biology Laboratory, National Institute of Oncology, 1122 Budapest, Hungary

**Keywords:** PDX, patient-derived xenograft, cancer therapy resistance, acquired resistance, tumor heterogeneity, targeted therapeutic strategies

## Abstract

Cancer accounts for approximately ten million deaths annually. The majority of these are attributable to resistance-driven tumor progression and metastasis. Although increasingly effective, precise, and selective therapeutic strategies are being developed, cancer cells retain the capacity to dynamically alter their phenotype and evade treatment. Traditional in vitro approaches rely heavily on cell line monocultures; however, their limited clinical translatability has driven the development of more advanced model systems. Three-dimensional in vitro models, including spheroids, organoids, and bioprinted tissues, provide more physiologically relevant and rapid insights, but fail to capture systemic pharmacodynamics and anatomical complexity. Emerging in vivo models, such as genetically engineered mouse models (GEMMs) of carcinogenesis and patient-derived xenografts (PDXs), as well as their derived organoids, provide a more comprehensive understanding of tumor biology. The preservation of tumor heterogeneity, microenvironment, and drug sensitivity profiles has positioned PDX models as widely used platforms in both drug development and therapy response prediction. Despite limitations—including variable engraftment rates, genetic drift, lack of fully functional immune systems, ethical concerns, and high costs—PDX models, when integrated with complementary techniques, contribute significantly to identifying novel therapeutic targets and combinations. Moreover, they support clinical decision-making by enabling drug response prediction based on genetic landscapes and co-clinical response data.

## Simple Summary

Therapy resistance is a major challenge in cancer treatment, and research aimed at overcoming resistance needs reliable, reproducible, and translatable preclinical models. This review summarizes widely used in vitro (2D cell line cultures, co-cultures, 3D models such as spheroids, organoids, and bioprinted tissue constructs) and in vivo models (e.g., allograft, xenograft, humanized, and genetically engineered), highlighting both their advantages and limitations. Special emphasis is placed on patient-derived xenograft (PDX) models, which have recently emerged as a widely accepted gold standard of drug efficacy and tumor sensitivity testing. We discuss the growing role of PDXs in drug resistance research, presenting recent advances across multiple tumor types.

## 1. Background

Cancer is among the leading causes of death worldwide [1]. Besides surgical removal of the malignant mass of solid tumors, systemic chemotherapy and radiotherapy are widely used. Hematological malignancies, or solid tumors with metastatic sites, need treatment that is not limited to a local region. Thus, the rapid development of different cytotoxic drugs against dividing cells was followed by a broad range of targeted therapy development, as key molecules were identified [2]. Furthermore, modalities that aim to enhance the host response to tumor presence, such as immunotherapies, are successfully used in clinical practice [3].

While our knowledge of cancer biology and potential compounds to attenuate tumor growth is intensively growing, some fundamental challenges remain to be addressed. Notably, about two-thirds of cancer-related deaths are associated with therapy resistance and the consequential metastatic spread [4]. While anticancer agents prove to be efficient, resistance can arise either from pre-existing genetic or phenotypic traits (intrinsic resistance) or from adaptive changes induced by treatment pressure (acquired resistance). In both scenarios, therapy failure and lack of treatment options are unavoidable [5].

Therapy resistance has been extensively studied using a broad spectrum of model systems, ranging from in silico approaches and in vitro cell line studies to advanced 3D culture systems and in vivo animal models. The emergence of genetically engineered mouse models (GEMMs) and patient-derived xenograft (PDX) models has significantly enhanced our understanding of resistance mechanisms. These systems provide insights that were previously unattainable and offer new opportunities to overcome therapy failure. This review focuses on the application of various model systems—particularly PDX models—in the study of cancer therapy resistance.

## 2. Cancer Therapy Resistance

Despite significant advances in cancer treatment, including targeted small-molecule inhibitors (e.g., tyrosine kinase inhibitors), biologics (e.g., monoclonal antibodies), immunotherapies, and personalized approaches such as gene therapy or chimeric antigen receptor (CAR)-T cell-based treatments, drug resistance remains a major challenge in advanced cancers. Resistance to anticancer therapies is a principal driver of treatment failure and disease progression, as defined by Response Evaluation Criteria in Solid Tumors RECIST-iRECIST criteria. This ultimately contributes to more than two-thirds of cancer-related deaths [4].

Cancer therapy efficacy reflects the interaction between the drug, the tumor, and the host microenvironment [6].

Tumor progression can significantly influence therapeutic effectiveness by altering drug accessibility, modifying the tumor microenvironment (including pH, oxygenation, stromal cell content, extracellular matrix composition, cytokines, and metabolites), and affecting systemic physiological conditions such as drug metabolism and immune responses. Effective systemic therapies typically induce tumor shrinkage through cell death, consistent with the Norton–Simon hypothesis [7].

However, tumor heterogeneity plays a critical role in enabling survival under therapeutic stress. As discussed later, this heterogeneity is a key reason for the limited clinical relevance of traditional cell line-based models, which rely on clonally selected, rapidly proliferating populations [8].

Biological barriers also impede effective drug delivery. The blood–brain barrier is a well-characterized example, limiting drug penetration into the central nervous system. At the molecular level, ATP-binding cassette transporters—including multidrug resistance (MDR) proteins—play a role in drug efflux and are frequently overexpressed in resistant tumors [9]. Additional physical barriers may arise within tumor tissues due to irregular vascularization and structural features.

The immune system is a critical component in tumor control. Cancer cells can actively reshape the tumor microenvironment to suppress immune responses, forming the basis for modern immunotherapeutic approaches [10,11].

Target molecules can change or become downregulated to evade drug effect [12]. Furthermore, bypassing routes [13,14], or constantly activated downstream effectors [15] also undermine drug effect.

The distribution and metabolism of the anticancer agents are key features for therapy success, and medicine activation [16] and ability to reach the tumor cells [17] should be promoted for therapy success.

**Figure 1:**
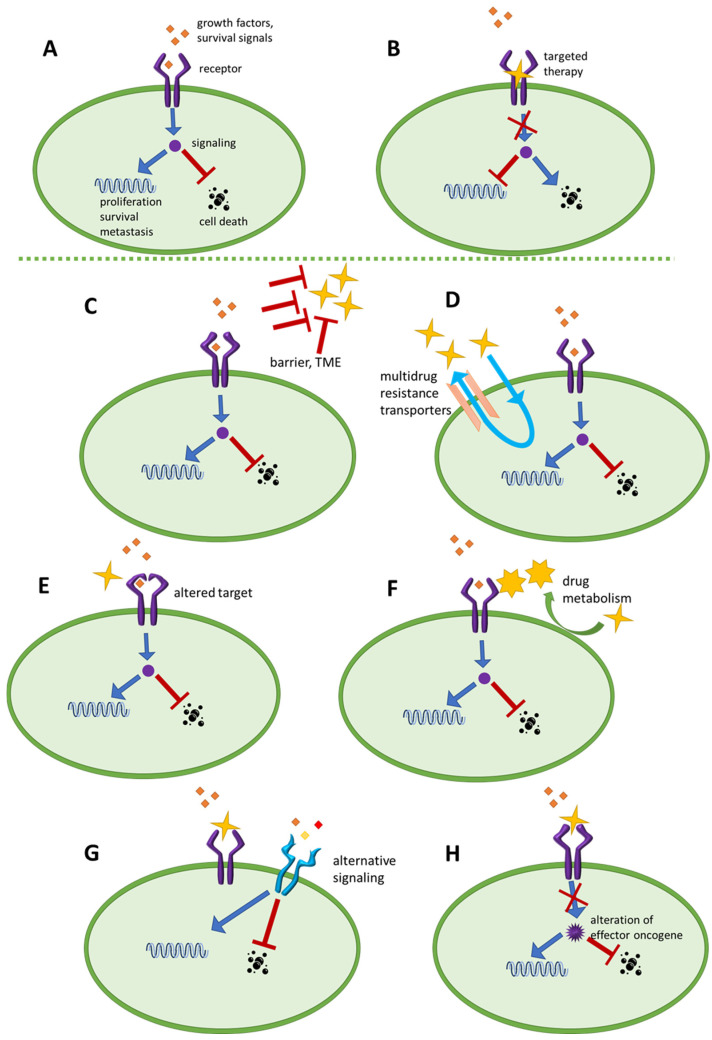
Routes of cancer therapy resistance in cancer cells. Cancer cells exhibit extended proliferative and metastatic activity (**A**), which can be disrupted by effective therapies and cell death can be achieved (**B**). Drug efficacy can be avoided by physical barriers surrounding the cells (**C**); exclusion of the drug by MDR transporters (**D**); alteration of the target molecule or the compound itself (**E**,**F**); bypassing the drug-induced blockade of a signal (**G**); reactivation of downstream signaling independently from the growth signal (**H**).

The major mechanism types behind therapy failure are summarized in Figure 1.

Resistance mechanisms are multifactorial and include pathway reactivation, compensatory signaling, and phenotypic plasticity. For example, in B-Rapidly Accelerated Fibrosarcoma gene (BRAF)-mutant melanoma, targeted inhibition of BRAF V600E frequently leads to rapid resistance due to activation of alternative signaling pathways. Understanding these escape mechanisms is essential for developing effective combination therapies and improving clinical outcomes [18].

Overcoming therapy resistance remains one of the central challenges in oncology. Advances include targeting previously considered “undruggable” molecules such as Rat Sarcoma (RAS) gene [19], exploiting synergistic drug combinations [20], and developing innovative delivery systems (e.g., liposomes, nanocarriers) to enhance drug accumulation and avoid efflux-mediated resistance [21]. Emerging approaches also target tumor metabolism, angiogenesis, epithelial–mesenchymal transition (EMT), and other adaptive processes. Co-clinical trials and AI-driven methods [22] further support treatment optimization by enabling rapid evaluation of drug combinations and dosing strategies.

Although many chemotherapeutic and targeted agents are capable of eliminating the majority of tumor cells, tumor repopulation remains a frequent outcome. Drug-tolerant persister (DTP) cells may arise from various subpopulations of cancer cells within a given clinical scenario. While certain expression patterns may predispose to the development of the DTP phenotype, it is increasingly evident that persistence is not restricted to a predefined subset of tumor cells. Rather, environmental selection pressures likely contribute to the emergence of these “surviving” cells, and their genetic or phenotypic repertoire determines the key molecular drivers of resistance and survival [23]. While total tumor eradication remains rare due to phenomena such as dormancy and metastasis [24], long-term disease control is increasingly achievable. The dynamic nature of resistance highlights the necessity for equally adaptable therapeutic strategies that address both genetic and epigenetic determinants [25,26].

All cancer research needs effective preclinical model systems. So far, the high attrition rate at the clinical phase is a major concern [27]; therefore, the introduction of novel models was necessary. At present, we are equipped with a broad selection of model choices both in vitro and in vivo.

## 3. Preclinical Cancer Models

### 3.1. In Vitro Models

Cell line–based research remains a cornerstone of biomedical and translational science, offering controlled, reproducible, and scalable in vitro systems for investigating cancer biology, molecular signaling, drug discovery, and personalized medicine. These systems inherently reduce the ethical and logistical constraints associated with animal experimentation [28]. The integration of high-throughput screening (HTS) technologies has enabled simultaneous evaluation of thousands of compounds across cell panels for target discovery and validation [29]. Large-scale multiomic datasets such as the Cancer Cell Line Encyclopedia (CCLE) and Genomics of Drug Sensitivity in Cancer (GDSC) have enabled systematic analysis of signaling pathways and gene regulation in cancer cell lines [30,31]. Similarly, the National Cancer Institute’s NCI-60 panel provides an extensive dataset of drug–response interactions across 60 human tumor cell lines [32]. CRISPR-based genome-wide screening approaches have further refined the identification of synthetic lethal interactions, wherein the simultaneous inhibition of specific gene pairs leads to selective cancer cell death. Notably, these approaches have uncovered vulnerabilities such as poly (ADP-ribose) polymerase (PARP) dependency in breast cancer gene (BRCA)1/2-deficient tumors, enabling the development of rationally designed targeted therapies [33].

A classic example of successful combination therapy is the dual inhibition of BRAF and MAPK/ERK kinase (MEK) in BRAF V600E-mutant melanoma. While single-agent BRAF inhibition induces significant tumor regression, resistance frequently emerges due to reactivation of the mitogen activated protein kinase (MAPK) signaling pathway. This observation led to the clinical adoption of combined BRAF and MEK inhibition, which significantly improves progression-free and overall survival compared to monotherapy [34]. Nevertheless, resistance remains a persistent challenge even in combination regimens [35].

Despite these advantages, conventional two-dimensional (2D) monolayer cultures remain limited in their ability to replicate in vivo tumor biology. They fail to recapitulate tissue architecture, cellular heterogeneity, and the complexity of the tumor microenvironment, thereby reducing their predictive value and contributing to poor clinical translation. Genetic drift under prolonged culture conditions has been demonstrated at the genomic level, further limiting reproducibility [36]. Cells cultured on rigid, planar surfaces adopt non-physiological morphologies, display altered gene expression profiles, and lack critical cell–cell and cell–extracellular matrix (ECM) interactions [37]. Additionally, phenotypic instability and the accumulation of culture-induced mutations compromise their biological relevance [38,39]. Therapeutic discrepancies further highlight these limitations. For instance, BRAF inhibition leads to rapid feedback activation of epidermal growth factor receptor (EGFR) signaling in colorectal cancer (CRC), but not in melanoma. This difference explains the limited clinical efficacy of BRAF inhibitor monotherapy in CRC and supports the rationale for combined EGFR and BRAF inhibition [40]. However, clinical studies revealed that EGFR expression alone does not reliably predict therapeutic response, emphasizing the complexity of resistance mechanisms and the limitations of simplified models [41]. Clinical benefit was ultimately achieved through triple combination strategies targeting BRAF, MEK, and EGFR [42]. Such cases urged the transition to more relevant models.

To address these limitations, advanced 3D culture systems—including spheroids, organoids, and bioprinted tissues—have been developed to better replicate tissue-level architecture and function [43]. Co-culture systems incorporating multiple cell types (e.g., tumor–stromal or tumor–immune interactions) provide additional insights into the tumor microenvironment (TME) [44]. Embedding cells within ECM-like scaffolds, such as collagen or matrigel, restores polarity, differentiation, and more physiologically relevant signaling networks. These systems also establish gradients of oxygen, nutrients, and drug penetration, which are critical determinants of therapeutic response [45]. Tumor spheroids, as multicellular aggregates, exhibit structural and metabolic heterogeneity and are therefore valuable tools for studying drug penetration and resistance mechanisms [46]. Patient-derived organoids (PDOs), generated from primary tumor tissue, have emerged as powerful platforms for precision oncology, allowing ex vivo drug testing tailored to individual patient profiles [47,48,49]. Notably, even traditional cell lines cultured in 3D configurations display gene expression patterns and drug sensitivities that more closely resemble clinical behavior [37,50]. 3D bioprinting enables spatial organization of multiple cell types and ECM components in defined architectures that mimic tissue or organ-specific architecture [51]. 3D bioprinted models can address complex issues like the metastatic process [52], TME [53], or tumor senescence [54], offering an ethically superior alternative to animal experiments [37].

The application of 3D culture systems has also enhanced the understanding of resistance to BRAF-targeted therapies. [55,56]. Experimental data indicate that hypoxic conditions within 3D systems can drive resistance to vemurafenib, and controlled 3D culture environments effectively model this phenomenon [28]. Comparative studies have demonstrated that 3D models more accurately capture resistance-associated gene expression profiles compared to 2D systems [57]. Additionally, co-culture systems incorporating stromal components further modulate drug sensitivity, emphasizing the critical role of the microenvironment in resistance biology [58].

Testing the effect of dabrafenib and trametinib on established PDOs from melanoma brain metastases, including BRAF V600E cases, showed differential sensitivity and utility of PDOs for predicting responses [59]. Melanoma PDOs carrying complex BRAF mutations (V600E; K601Q) were used to study BRAFi MEKi combination, showing the importance of Notch signaling [60], while BRAF V600E CRC PDOs display distinct metastasis activity based on glutathione synthesis activity [61]. Future directions will likely emphasize hybrid models that combine the scalability of traditional cell lines with the architectural and functional fidelity of 3D and patient-derived systems.

Despite these advances, in vitro models remain inherently limited by the absence of systemic physiological factors, including circulation, metabolism, and immune responses. These limitations restrict their ability to accurately predict whole-organism drug effects. Nevertheless, in specific applications such as high-throughput drug screening, the advantages of in vitro systems often outweigh their constraints.

### 3.2. In Vivo Models

In vivo models remain indispensable in cancer research, as they provide a more comprehensive representation of tumor biology, including vasculature, extracellular matrix composition, and immune system interactions. These models enable the assessment of pharmacokinetics and pharmacodynamics, which cannot be fully replicated in vitro. Moreover, drug resistance mechanisms related to the microenvironmental factors are not visible in cell cultures [62,63]. Furthermore, they allow investigation of metastasis formation and therapeutic efficacy in suppressing disease progression [64].

A diverse range of in vivo cancer models has been developed, including cell line–derived allografts (CDA), xenografts (CDX), genetically engineered mouse models (GEMMs), patient-derived organoid xenografts (PDOX), and patient-derived xenografts (PDX).

Syngeneic allograft models utilize immunocompetent mice, enabling the study of immune-mediated effects on tumor growth and therapeutic response [65,66]. These models are cost-effective and reproducible but are limited in their ability to recapitulate human tumor biology.

In contrast, xenograft models involve the transplantation of human tumor cells or tissues into immunocompromised animals [62]. For this purpose, many different mouse strains have been developed. As concluded recently [67,68], immunocompromised mouse strains are needed for the successful engraftment of human cells or tissues. Nude [69], severe combined immunodeficiency (SCID) [70], non-obese diabetic SCID (NOD-SCID) [71] and NOD-SCID-gamma (NSG) [72] mouse strains are well-known for hosting, which exhibit varying degrees of immunodeficiency (Table 1).

More susceptible host strains are more prone to spontaneous lymphoma, and partial leakage in T-/B-cell functions after knockout is also to be considered when choosing the appropriate model for PDX experiments. Also, immunodeficient mice lack immune response, limiting their use in the investigation of immune checkpoint inhibitor therapies or other immunotherapeutic modalities. Thus, the use of humanized mice is quickly developing. Humanized mice originate from NSG (NOD.Cg-*Prkdc*scid *Il2rg*tm1Wjll/SzJ) [72], NOG (NOD-Cg-*Prkdc*scid *Il2rg*tm1Sug/JicTac) [73] or BRG (C.Cg-*Rag1*tm1Mom *IL2rg*tm1Wjl/SzJ) mice [74].

The tumor-infiltrating lymphocytes (TILs) and peripheral blood mononuclear cells (PBMCs) are classically used for the generation of humanized mice, producing mainly human NK cells and major histocompatibility complex (MHC)-restricted T cells. Graft versus host disease is typical, terminating such experiments after 2–5 weeks. Additionally, fully humanized models are also developed, using hematopoietic stem cells (HSCs) to generate B cells and MHC-restricted T cells, or other immune components (monocytes, macrophages, neutrophil granulocytes, dendritic cells). This technology can be applied in genetically engineered strains expressing human cytokines that drive immune cell maturation, such as models NOG-GM3, NSG-SGM3, and MISTRG, to further improve human immune system modeling. These models are already extensively reviewed [75].

Cell line-derived tumors exhibit reduced heterogeneity due to adaptation and clonal selection, limiting their ability to faithfully model clinical tumors [76,77].

In contrast, GEMMs allow tumor development within a defined genetic context, closely mimicking oncogenic processes. However, these models often produce relatively homogeneous tumors and may not accurately reflect metastatic behavior [78]. This strategy, given the extent of applicability of modern genome editing tools [79], has huge potential, but leads to more uniform tumors, and often lacks the ability of GEMMs to form metastasis in vivo [80].

Patient-derived xenograft (PDX) models overcome these limitations. The engraftment of surgical tumor tissue forms various PDXs. If in the first step, in vitro PDOs are generated, they can be subsequently xenografted (PDOX) as well. As PDX models preserve clinical microenvironment, heterogeneity, and structural traits, they serve as a reliable model of cancer progression, drug response and drug resistance, or metastasis formation. The establishment of different cancer types into PDXs is well documented [68]. This great clinical translatability is not only theoretical: it is proven that the drug response of PDX models closely resembles that of clinical tumors [81].

PDX models are widely spreading worldwide: their reliability and complexity seem to overweigh their financial and time requirements. The use of PDX models in novel drug evaluation is now a general standard, promising a higher rate of translatable results. Of note, National Cancer Institute’s Patient-Derived Model Repository (PDMR; [82]) was established in 2012, and gradually overtook as standard platform for drug screening rather than the long-history NCI-60 cell line panel screen.

## 4. PDX in Cancer Therapy Resistance Research

Cell line-based models offer high reproducibility and experimental control; however, they are inherently limited to a narrow subset of cancer cells. In contrast, more heterogeneous systems, such as spontaneous animal tumors, their derived allografts, or patient-derived xenografts, enable the simultaneous investigation of multiple cellular lineages. This enables the identification of resistance mechanisms that may not arise or be maintained under in vitro conditions, but are present in more complex systems and in patients as well. Numerous studies have reported resistance mechanisms across different cancer types. Although these studies have employed a variety of model systems—from in vitro cell lines to patient-derived samples—they revealed a diverse spectrum of resistance drivers, as previously reviewed [23]. These findings are partially recapitulated in PDX models and supported at the clinical level through the analysis of clinicopathological samples.

PDX models can be established at multiple time points, from different anatomical sites, and at various stages of tumor evolution. Consequently, they have become widely adopted in oncology research, particularly in drug development. In the context of therapy resistance and tumor progression, PDX models provide additional advantages by harboring potential diverse resistance mechanisms, particularly those that cannot be sustained under in vitro growth conditions.

Three main approaches characterize the use of PDX models in resistance research. In co-clinical “avatar” trials, patient-derived tumors are engrafted into mice at surgical removal and exposed to multiple therapeutic regimens simultaneously. This allows for the identification of the most effective treatment strategy, potentially even before disease progression occurs in the patient. While this approach offers direct translational relevance, it is time-consuming and generally limited to later-line therapeutic decisions, focusing on individual patients.

A second approach involves generating PDX models from treatment-naïve tumors and from tumors obtained after relapse or resistance. This strategy may include the collection of metastases from multiple anatomical sites, enabling the investigation of spatial, temporal, and therapy-induced evolution of tumor genetics. Such models are valuable for identifying novel therapeutic targets aimed at preventing or reversing resistance. Large-scale PDX collections, such as the Novartis Institutes for Biomedical Research PDX Encyclopedia (NIBR PDXE), provide valuable datasets linking genetic profiles to therapeutic response patterns [81].

The third approach is conceptually similar to the first, but focuses on longitudinal treatment of established PDX models. Continuous therapy is applied to mimic clinical treatment regimens, allowing for the observation of acquired resistance over time. Unlike in vitro or cell line-derived xenograft systems, where resistance may develop rapidly, PDX models often require extended observation. For example, in our previous study on BRAF V600E-mutant melanoma treated with continuous vemurafenib, resistance development was monitored for approximately 600 days [83]. Such long-term studies introduce specific challenges, including age-related comorbidities in host animals and the need for sustained experimental management. Therefore, comprehensive documentation of all experimental variables, including unforeseen events, is essential to enable reproducibility and facilitate the accumulation of meaningful correlative data.

While PDXs are widely used, all specimens harbor traits unique to the given patient tumor. Most academic laboratories use their own tumor models to establish their hypotheses—models that are often difficult for others to access and reproduce. For-profit PDX biobanks offer greater reproducibility, but at a high cost. Therefore, there is an urgent need to characterize the exact models used and to publish all relevant data to enable meaningful comparison with those reported by others. In the following, we look at several major cancer types to investigate recent advances in understanding resistance mechanisms using the PDX toolkit.

## 5. Achievements Using PDX Models in Different Cancer Types

### 5.1. Prostate Cancer

Broad panels of prostate cancer (PCa) PDXs were introduced for drug efficacy studies. The MD Anderson collection (MDA PCa PDX) consists of 80 PDX models from 47 patients [84], while the Melbourne Urological Research Alliance (MURAL) collection offers 59 PDXs from 30 patients [85]. These extensive series aim to cover much of the genetic landscape variation among patients with prostate cancer, from treatment-naïve to metastatic, castration-resistant cases, with diverse genetic backgrounds. Additional public PDX repositories have also been described previously, as reviewed in [86]. It has also been shown that, so far, only a fraction of available models are represented in these repositories, while the majority of established PDXs remain restricted to local academic use and private collaborations. Therefore, the Movember GAP1 (Global Action Plan 1) initiative compiled an international collection of academic prostate cancer PDX models maintained in different laboratories, together with genetic characterization and contact information to facilitate further use [87]. These continuously expanding collections of models and the corresponding datasets reveal the genetic background of tumors paired with the clinical outcomes of the patients. As standard protocols are being developed [88], all of these results will become more comparable. Rare subpopulation genotypes are effectively characterized [89], and screened for therapy response.

### 5.2. Non-Small Cell Lung Cancer (NSCLC)

The first molecular therapy in lung cancer targeted the epidermal growth factor receptor (EGFR); since then, multiple generations of small-molecule inhibitors have been developed. It is known that EGFR mutation (L858R) or exon 19 deletion [Del19] can determine sensitivity; therefore, anti-EGFR therapy is preceded by the routine genetic testing [90]. It was shown that the EGFR T790M mutation mediates resistance to EGFR inhibitors gefitinib and erlotinib, while third-generation EGFR inhibitors, such as osimertinib, are also effective in T790M-positive tumors, although resistance can still develop through the EGFR C797S mutation [91]. Novel compounds have even been shown to overcome this triple alteration [92]. The fact that recent treatment advances increasingly involve patient-derived models underlines the importance of such systems in the study of anti-EGFR therapy resistance in NSCLC, both in monotherapy and in combination regimens [93]. Furthermore, non-EGFR-mediated resistance mechanisms have also emerged partly through the use of PDXs: for example, the role of AXL kinase in a feedback loop during osimertinib failure has been demonstrated [94]. Our recent study also evaluated the applicability of a liposomal formulation of a highly toxic anthracycline derivative, which proved to be safe and effective in a lung adenocarcinoma PDX model [95]. Recently, a CAR-T cell-based approach was proven to be effective against NSCLC drug-tolerant persister cells [96]. In another small cohort of lung cancer PDX models, acquired resistance against osimertinib evolved, and the characterization led to identification and successful application of additional ERK inhibitor therapy [97]. A broad collection involving 39 zebrafish PDXs of NSCLC showed ability to precise treatment prediction as quickly as in three days [98].

Importantly, a thorough analysis of NSCLC PDX models established from 19 clinical samples from 9 patients compared tumors at different stages of resistance in order to assess MET polysomy/amplification and the efficacy of osimertinib combined with the MET inhibitor savolitinib. This type of temporal evolution, which would otherwise remain largely inaccessible, can be followed effectively using a panel of related PDX models [99].

### 5.3. Colorectal Cancer

In colon cancer, alongside a novel shRNA synthetic lethality screening approach, a panel of KRAS-mutant PDX models demonstrated that MEK inhibitor treatment causes systematic upregulation of ERBB2 and ERBB3, thereby promoting proliferation, and that dual inhibition of EGFR and ERBB2 (such as with afatinib and dacomitinib) can act synergistically together with the MEK inhibitor selumetinib [100]. Similarly, another study found that, in metastatic CRC PDX models, combined inhibition of EGFR, HER2, and ERBB3 (trastuzumab + lapatinib) led to tumor eradication [101]. The investigation of 125 PDXs led to the discovery that IGF2 overexpression in a subset of patients can contribute to cetuximab resistance [102]. PDX experiments also serve multiple purposes: for example, it was shown that inhibition of ferroptosis leads to multidrug resistance, which can be experimentally reversed through Nrf2 (nuclear factor erythroid 2-related factor 2) [103]. In addition, expression of the multidrug resistance determinant P-glycoprotein (P-gp) was associated with a reduced tumor/plasma ratio of a p21-activated kinase inhibitor and with corresponding effects on tumor growth in a panel of 12 PDX models [104]. The novel target circular RNA circPDIA3 was also shown to play a role in oxaliplatin resistance in CRC models [105]. In a subgroup of PDX models, secondary resistance mechanisms have been followed to emerge [106].

Importantly, the different consensus molecular subtypes of CRC are well represented in PDX models, with a relatively high establishment rate of 65–89%, and these models also carry the potential to investigate microbiome-related effects, which may contribute to CRC development and progression [107]. Due to the large number of known driver mutations and inherited syndromes such as familial adenomatous polyposis, GEMM are also commonly used in CRC studies [108], as are induced carcinogenesis models using 1,2-dimethylhydrazine or azoxymethane [109], completing the landscape formed by PDX experimental studies.

### 5.4. Breast Cancer

The establishment rate of breast cancer PDXs is lower (25% from primary tumors and 36% from metastatic lesions), particularly for estrogen receptor-positive (ER+) models, which are more difficult to establish and require hormone supplementation in the host animals. In contrast, triple-negative breast cancers (TNBC), which lack ER, PR, and HER2 expression, show a higher success rate, reaching 58% from primary tumors and 85% from metastatic lesions. Paired patient samples, PDXs, and PDOXs have been analyzed across hundreds of samples, and highly conserved biological features have been demonstrated. This conservation applies to gene expression and mutational status, as well as to extended drug screening results, and some of these combined findings have already been translated back into clinical decision-making for patients. This illustrates how, although with considerable effort, a well-developed preclinical system can directly benefit patient care [110]. A number of key genes have been identified in therapy resistance, including CCNE1 amplification in resistance to CDK4/6 inhibitor therapy [111], persistent ER activity after clinical use of fulvestrant, which supports improved outcomes with the novel ER inhibitor vepdegestrant [112], and lysyl oxidase (LOX), an extracellular matrix-remodeling enzyme induced by hypoxia that promotes chemoresistance and may therefore serve as a suitable resensitizing target in breast cancer therapy [113]. In the field of CDK4/6 inhibitor resistance, a library of 26 luminal breast cancer PDX models was established and revealed the role of proliferative pathway members such as MTORC1, E2F, and MYC [114]. Palbociclib-resistant PDX and organoid models showed sensitivity to abemaciclib, which might lead to a new biomarker-driven therapy option [115].

### 5.5. Ovarian Cancer

Platinum resistance is a major cause of therapy failure in ovarian cancer and therefore needs to be controlled or overcome. Recently, a combination of the polo-like kinase-1 (PLK1) inhibitor onvansertib with either gemcitabine or carboplatin was found to be effective in PDX models with either intrinsic or acquired resistance to cisplatin [116]. As for cisplatin resistance, its key metabolic changes were identified and successfully repressed by combined metformin-cisplatin regime in PDXs [117]. A zebrafish PDX system has also been introduced for screening carboplatin response [118]. Furthermore, the mechanism underlying ferroptosis resistance has also been described [119]. Alteration of homologous recombination has been proposed as a strategy to overcome platinum resistance [120]. Resistance to PARP inhibitors has also been investigated, and secondary inhibition approaches have been tested successfully [121]. Of note, the success rate of model establishment varies considerably depending on inoculation site, host mouse strain, and the stage and aggressiveness of the implanted tumors [122]. This highlights the need for more sophisticated engraftment procedures in order to reflect the clinical situation of most patients with ovarian cancer more accurately. Recently, large scale ovarian cancer PDX biobank characterization was reported, including evolution of resistance in xenograft state, and identifying changes in interferon response, Wnt-beta-catenin signaling, EMT, cell cycle or inflammatory response [123].

### 5.6. Malignant Melanoma

Therapy resistance in melanoma is a fundamental issue, as it occurs in the majority of patients. Rapid tumor growth and a highly metastatic phenotype leave most patients in need of systemic therapy. Response rates and patient survival have improved substantially owing to recent therapeutic advances; however, melanoma continues to exhibit a wide range of primary, secondary, and immune-related resistance mechanisms [18]. Although the methodologies underlying the accumulated data are diverse, it is evident that more recent studies increasingly incorporate PDX-derived findings. The BRAF V600E mutation, present in approximately 50% of patients, represents a highly druggable target. Mutation-specific drugs were introduced beginning in 2011 (vemurafenib), later followed by dabrafenib and encorafenib in clinical use. However, the rapid development of resistance soon highlighted compensatory activation of the mitogen-activated protein kinase (MAPK) pathway, leading to the successful clinical implementation of combined BRAF and MEK inhibition (cobimetinib, trametinib, binimetinib) [124]. Nevertheless, resistance ultimately emerged even against this dual blockade [125]. PDX models have been used to examine resistant clones [126], and the analysis of large-scale collections has described the variation in the genetic landscape of melanoma [127], offering secondary therapies that reverse (preprint data) [128] or overcome dual resistance [129]. PDXs have also been shown to preserve genetic concordance with the original tumors [130], and they have even supported the demonstration of anti-HER2 CAR-TIL (chimeric antigen receptor–tumor infiltrating lymphocyte) therapy efficacy [131]. In addition, PDX studies have revealed that metabolic alterations [132] and non-coding RNAs [133] may play crucial roles in drug sensitivity. In our previous work, which involved long-term BRAFi treatment in vivo with sequential sample collection and transcriptome analysis, we found that none of the canonical resistance-associated changes occurred, despite the clear appearance of in vivo resistance. Instead, we identified novel candidate players, including ABCB1, as a potentially druggable determinant of resistance [83]. Furthermore, our PDX model supported the development of a novel therapeutic strategy based on the modulation of cysteine metabolism [134]. Others have emphasized that each PDX follows its own trajectory on the map of drug-induced evolution [135,136], although certain recurrent mechanisms can still be identified by comparing genomics of therapy-naïve and therapy-resistant PDXs derived from the same patients, in which case the evolutionary process itself had already taken place in the patients [137]. As an additional challenge, intratumoral and intertumoral heterogeneity should be addressed and monitored more precisely [138,139]. Melanoma PDX models also closely resemble their clinical counterparts and can therefore function as “xenopatients” to support medical decision-making [140].

### 5.7. Kidney Cancer

In renal cell carcinoma (RCC), both tyrosine kinase inhibitor (TKI) therapies and immunotherapies are commonly applied. A large number of studies have identified resistance mechanisms using traditional model systems, such as cell lines and CDXs [141], but only few have reported resistance mechanisms based on PDX studies [142,143]. In our experience, this may be due to the time-consuming nature of this study design: in clinical practice, patients typically undergo surgery, and then relapse may occur only years later, at which point targeted therapy becomes necessary and may keep the tumor in remission for another 5–10 years. One report showed that low engraftment success rates and a bias toward more aggressive, metastatic, and resistant models are characteristic of RCC PDX systems [144]. Importantly, a broad PDX cohort of 46 RCC models was established and characterized for standard of care therapy response, to provide basics of PDX-driven efficacy and resistance research in RCC [145].

### 5.8. Pancreatic Cancer

Being often surgically irresectable, aggressive and therapy-resistant, pancreatic cancer is a major challenge to the cancer research community. However, PDX models are intensively used to find new ways. A subpopulation of cells with viral mimicry response hyperactivation expressed trametinib resistance, which could be overcome by co-inhibition of MAX dimerization factor 1 [146]. The brand new targeted combination of KRAS (daraxonrasib), EGFR family (afatinib) and STAT3 (SD36) proved on a panel of pancreatic cancer PDX models to express antitumor activity without emergence of resistance for prolonged time [147]. Furthermore, combined treatment with silmitasertib was found to restore gemcitabine sensitivity in pancreatic cancer PDX models [148]. BRCA1/BRCA2/PALB2 altered cancer PDX panel of pancreatic, breast and ovarian origin concluded that the PARP inhibitor saruparib alone or in combination with chemotherapy had prolonged antitumor effect and evaded acquired olaparib resistance [149]. These recent advances represent a new horizon in pancreatic cancer maintenance.

## 6. Limitations and Recent Developments

PDX models have proven exceptionally useful in the study of cancer therapy response. However, while their heterogeneity allows them to resemble clinical tumors more closely, it also introduces the possibility that different regions of the tumor may grow, evolve, and behave differently. Although this criticism remains valid, the fact is that the 1 × 1 × 1 (one patient, one drug, one mouse) model design has shown high reliability [81]. On the other hand, single-cell sequencing and spatial biology approaches may provide deeper insight into how representative a given tumor tissue sample truly is. Such studies have already been published [150], suggesting that limiting generation number or using multiple parallel PDX models can provide a more complete picture, while also raising the question of whether tumor evolution follows the same pathway each time from the same origin. In our view, although this phenomenon must be considered carefully to avoid overgeneralization, it still reflects natural biological processes and yields clinically relevant results.

Another major challenge is the success rate of engraftment. Tumor establishment rates vary widely, typically being low for early-stage primary tumors and high for samples derived from metastatic lesions. Studies have reported success rates ranging from 5% to nearly 100%, consistently showing higher rates for advanced metastatic lesions, often associated with specific molecular backgrounds, as reviewed in breast cancer studies [114,151]. These features may lead to a skewed population of derived PDX models, which in turn may produce findings that are not fully representative of the broader patient population. Similarly, small early-stage tumors are generally not available for research use because they are reserved for diagnostic purposes, making the earliest phases of tumor evolution difficult to study using PDX models. While these limitations are unavoidable, the value of PDX models in resistance mechanism discovery and drug sensitivity studies remains substantial. However, population-level conclusions should not be drawn based on collections of PDX models only.

Immunotherapy has become a major hope in cancer treatment. Immune checkpoint inhibitors, CAR-T cell therapies, monoclonal antibodies, cytokines, and various cancer vaccines are all now established in clinical practice. While conventional immunocompromised mice provide little or no opportunity to investigate such therapies, humanized mice can serve as valuable platforms in these studies, and it has been reported that findings obtained in such PDX models are concordant with clinical outcomes [152].

An important additional issue is tumor heterogeneity and diversity over time and across host animals. For this reason, biomarker studies are not ideally performed in PDX models, because the model itself is continuously changing and evolving. During serial passaging and tumor growth, human components of the tumor microenvironment are gradually replaced by murine elements, which may further drive tumor evolution [153]. To address this issue, there is an urgent need for standardized omics-wide profiling and single-cell-level analysis of these tumors in order to ensure adequate characterization. Selective pressure within the host animal is likely even stronger when, as in the majority of cases, tumors are implanted subcutaneously rather than orthotopically. In summary, careful documentation and publication of such characteristics are more important than ever. Unfortunately, a 2019 meta-analysis found that more than half of the evaluated studies provided insufficient validation data for the PDX models they used [154].

As many laboratories develop and use their own PDX models, it is nearly impossible to standardize methodology, passage history, genetic drift, or other confounding factors across studies. One possible solution is the broader use of international PDX repositories or biobanks that apply reliable and transparent pipelines for testing drug candidates. Several of these biobanks have been summarized in previous reviews [68,155]. Good practice can also be promoted through publicly available standard operating procedures, as exemplified by the NCI’s PDMR collection [82].

Limited time may also be a major obstacle when clinicians need to make treatment decisions before a fully developed xenopatient PDX becomes available. Although patient-derived organoids still show some discordance in drug response, a novel Mini-PDX model has been developed. In this system, tumor suspension is loaded into a hollow-fiber capsule, implanted into the mouse, treated in vivo, and typically removed after 7 days for assessment of cell viability within the capsule [156].

Finally, and importantly, animal experimentation—particularly in mammals—raises major ethical concerns. As recently reported, the National Institutes of Health stopped supporting new grant proposals based solely on animal models in 2025. This change encourages the integration of non-mammalian in vivo models, in vitro systems such as PDOs, and AI-driven early genomic prioritization of animal studies, possibly including Mini-PDX. This integrated strategy has a dual advantage: it provides multilevel data from multiple experimental platforms and reduces animal use. This concept has been referred to as PDX 2.0, describing a workflow that yields more reliable data while involving fewer animals [157].

**Figure 2:**
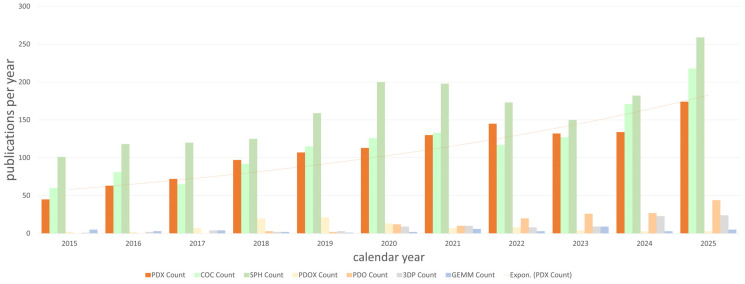
Number of yearly cancer resistance-related PubMed-indexed publications (2015–2025), by model. PDX: patient-derived xenograft; COC: coculture; SPH: spheroid; PDOX: patient-derived organoid xenograft; PDO: patient-derived organoid; 3DP: 3D printed model; GEMM: genetically engineered mouse model. Expon.: exponential linear regression fit on PDX count data.

Despite these limitations, the importance of novel models, including PDXs, is increasing dynamically. Using the PubMed literature database, the search query ‘cancer therapy resistance’ was combined with terms such as ‘PDX’, ‘coculture’, ‘spheroid’, ‘PDOX’, ‘PDO’, ‘3D printed’, and ‘GEMM’ [158]. An exponential trendline was fitted to the publication counts obtained for PDX-related articles (Figure 2). As shown, PDX, coculture, and spheroid are among the terms that have increased dynamically over time in association with cancer therapy resistance. Interestingly, GEMM and 3D-printed models did not show the same trend, possibly because they are more frequently used for other aspects of cancer research.

## 7. Summary

**Figure 3:**
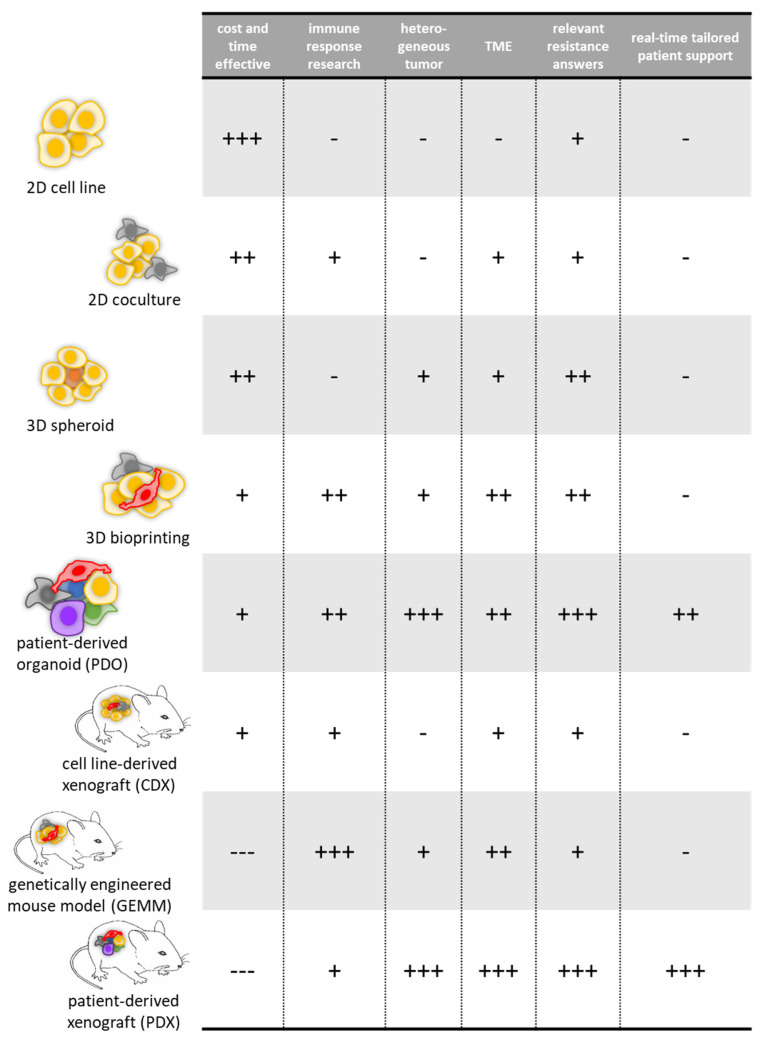
Advantages and challenges of different preclinical models of cancer drug resistance.

As cancer incidence continues to rise over time, there is a growing need to manage patients over prolonged periods while maintaining a high quality of life. Cancer cells are capable of escaping otherwise effective therapies by developing drug resistance. Preclinical models and clinical data have been studied extensively for decades in an attempt to decipher resistance mechanisms. However, even when one resistance mechanism is successfully targeted and a secondary therapy is applied, the process often restarts, leading cancer cells toward further escape routes. To address this phenomenon, which leads to progression, metastasis, and high mortality, preclinical models must be further optimized. Beyond the classical approach of studying 2D cancer cell cultures, a broad range of increasingly complex in vitro systems is now available, including co-cultures, 3D spheroids, organoids, tissue slices, 3D-bioprinted tissues, and lab-on-a-chip models, all further strengthened by advances in genome editing technologies. In our opinion, these fast, effective, and relatively inexpensive approaches should continue to be supported, but they must be complemented by in vivo animal studies in order to evaluate complex systemic processes such as adsorption, distribution, metabolism, and elimination (ADME) of anticancer treatments. Furthermore, as tumor heterogeneity has been shown to conceal clinically relevant resistance mechanisms from studies relying only on isogenic cancer cell clones, this clinical heterogeneity should be recapitulated as much as possible at the preclinical level. Patient-derived xenografts—and, for in vitro applications, patient-derived organoids—have therefore become highly popular and widely used tools in preclinical drug screening, resistance mechanism research, and the generation of valuable datasets for future drug development. Model characteristics are summarized in Figure 3.

As one of the most prominent signs of this paradigm shift, the National Cancer Institute, after 25 years and more than 100,000 compounds tested, discontinued active drug screening on the NCI-60 panel of 60 cell lines of different tissue origins and shifted its screening strategy toward hundreds of PDX models as a routine standard method [159]. A major challenge moving forward will be the standardization and proper comparison of these studies, since, unlike isogenic cell lines, all such models are unique. Genome-wide sequencing and proteomic analyses, even at the single-cell level, are already available, although they remain resource-intensive. A consensus on the desired methodological standards may be essential for bringing together the strengths of individual studies.

In addition, xenopatient studies can provide real-time drug response data across entire panels of therapies, and can now effectively contribute to bedside treatment decisions. Such approaches are currently being investigated in dozens of clinical trials, as recently reported [160]. This is shifting the concept of chemotherapy away from one-size-fits-all protocols toward personalized precision medicine. Recent progress in the accessibility and transparency of PDX data, including initiatives such as the PDX Finder portal [161] and the EurOPDX data portal [162], provides a new level of complex data collection that benefits both basic research and translational medicine. While, for most scientific questions, there is a relatively clear hierarchy of model selection based on model characteristics [163], emerging resistance still lacks a standardized methodological pipeline. We suggest that 3D cellular analyses [164], patient-derived organoids, and PDX cohorts [165] seem the most reliable source of clinically useful data.

## 8. Conclusions

Cancer resistance is the principal obstacle to maintaining successful therapeutic effects in most cancer types. Although preclinical research on resistance has a long history, much of the available evidence is still based on clonally selected in vitro cell lines with uncertain clinical relevance. Over the past 10–20 years, many innovative model systems have emerged, ranging from 3D spheroids, 3D bioprinting, and organoids to more complex in vivo systems such as GEMMs, PDXs, and humanized mouse models. As shown, PDX is particularly well suited for the engraftment of human tumors while preserving heterogeneity, tumor microenvironment, and structural features. These tumors display a drug resistance landscape similar to that of the original tumor and also allow the investigation of resistance evolution in vivo.

Importantly, PDX models also have several limitations. Even in these complex systems, clonal expansion and selection occur, leading to genetic drift over serial passages. However, this does not contradict the fact that clonal selection may also be driven by anticancer treatment itself. The lack of an intact immune system in host animals limits the applicability of conventional PDX models in immunotherapy studies. Moreover, cytotoxic and targeted therapies may affect intratumoral immune components, which may introduce bias into such analyses. Initial sampling conditions and disease stage also influence the success rate of PDX engraftment, limiting the suitability of these models for population-wide studies.

PDX models are therefore highly valuable tools in cancer resistance research; however, they must be designed and interpreted with caution. Emerging scientific policies that encourage the combined use of animal and non-animal modalities may help address these limitations, as multilevel in vitro and in vivo data (PDX, PDOX, GEMM, Mini-PDX, etc.), supported by standardized genomic and proteomic analyses and AI-driven pattern recognition, promise to improve the reliability of preclinical evidence. Most importantly, close collaboration with clinical practice, access to real-world patient data, and systematic comparative analysis are, in our opinion, essential for building successful preclinical pipelines.

## Figures and Tables

**Table 1:** Genetic modifications and their effects on the immune system that are used in the generation of immunocompromised mouse strains widely used in cancer xenograft models.

Animal Genotype	Affected Target	Immune Cell Deficiency
Nude	loss of Foxn1	T-cell function loss
SCID	DNA-protein kinase loss of function mutation	reduced T and B cell level
NOD	NK cells	function loss of NK cells
IL-2	interleukin 2 gamma chain inactivation	T cells, B cells, NK cells impaired
RAG1/2	recombination activating gene	T and B cells impaired

## Data Availability

All data presented in this review article are publicly available.
